# The Role of JAK/STAT Molecular Pathway in Vascular Remodeling Associated with Pulmonary Hypertension

**DOI:** 10.3390/ijms22094980

**Published:** 2021-05-07

**Authors:** Inés Roger, Javier Milara, Paula Montero, Julio Cortijo

**Affiliations:** 1 CIBERES, Health Institute Carlos III, 28029 Madrid, Spain; julio.cortijo@uv.es; 2 Department of Pharmacology, Faculty of Medicine, University of Valencia, 46010 Valencia, Spain; paulamonmag@gmail.com; 3 Pharmacy Unit, University General Hospital Consortium of Valencia, 46014 Valencia, Spain; 4 Research and Teaching Unit, University General Hospital Consortium, 46014 Valencia, Spain

**Keywords:** pulmonary hypertension (PH), Janus kinase 2 (JAK2), signal transducer and activator of transcription 3 (STAT3)

## Abstract

Pulmonary hypertension is defined as a group of diseases characterized by a progressive increase in pulmonary vascular resistance (PVR), which leads to right ventricular failure and premature death. There are multiple clinical manifestations that can be grouped into five different types. Pulmonary artery remodeling is a common feature in pulmonary hypertension (PH) characterized by endothelial dysfunction and smooth muscle pulmonary artery cell proliferation. The current treatments for PH are limited to vasodilatory agents that do not stop the progression of the disease. Therefore, there is a need for new agents that inhibit pulmonary artery remodeling targeting the main genetic, molecular, and cellular processes involved in PH. Chronic inflammation contributes to pulmonary artery remodeling and PH, among other vascular disorders, and many inflammatory mediators signal through the JAK/STAT pathway. Recent evidence indicates that the JAK/STAT pathway is overactivated in the pulmonary arteries of patients with PH of different types. In addition, different profibrotic cytokines such as IL-6, IL-13, and IL-11 and growth factors such as PDGF, VEGF, and TGFβ1 are activators of the JAK/STAT pathway and inducers of pulmonary remodeling, thus participating in the development of PH. The understanding of the participation and modulation of the JAK/STAT pathway in PH could be an attractive strategy for developing future treatments. There have been no studies to date focused on the JAK/STAT pathway and PH. In this review, we focus on the analysis of the expression and distribution of different JAK/STAT isoforms in the pulmonary arteries of patients with different types of PH. Furthermore, molecular canonical and noncanonical JAK/STAT pathway transactivation will be discussed in the context of vascular remodeling and PH. The consequences of JAK/STAT activation for endothelial cells and pulmonary artery smooth muscle cells’ proliferation, migration, senescence, and transformation into mesenchymal/myofibroblast cells will be described and discussed, together with different promising drugs targeting the JAK/STAT pathway in vitro and in vivo.

## 1. Introduction

Pulmonary hypertension (PH) is defined as a group of diseases characterized by a progressive increase in pulmonary vascular resistance (PVR), which leads to right ventricular failure and premature death [1]. The term pulmonary hypertension is defined as a mean pulmonary artery pressure (mPAP) greater than 25 mmHg, measured at rest according to the guidelines issued by the European Society of Cardiology (SEC) and by the European Respiratory Society (SER) [2].

The clinical classification of PH is intended to categorize multiple clinical conditions into five groups according to their clinical presentation, pathological manifestations, hemodynamic characteristics, and treatment strategies [2]. The clinical classification may be updated when new data regarding the above features become available or when additional clinical entities are considered. A comprehensive version of the clinical classification is presented in Table 1.

Group 1 is known as pulmonary arterial hypertension (PAH), which may be idiopathic or caused by human immunodeficiency virus, liver disease, or congenital heart disease (PAH-CHD). Group 2 PH is related to the left side of the heart. Long-term high blood pressure and mitral valve disease are also associated with group 2 PH. Group 3 PH refers to chronic lung diseases (PH-CLD) and/or hypoxemia. Group 4 PH is due to chronic thrombotic and/or embolic disease. Group 5 includes various blood disorders, systemic disorders, metabolic disorders, and other conditions such as kidney disease [3].

Janus kinase type 2 (JAK2) and signal transducer and activator of transcription 3 (STAT3) have been reported to participate in processes directly related to pulmonary artery remodeling such as smooth muscle proliferation, endothelial dysfunction, and inflammation. These cellular and molecular process are common in several vascular diseases such as vasculitis, atherosclerosis, and PH [4,5].

PH comprises multiple molecular pathways that lead to vasoconstriction, the remodeling of the pulmonary arteries, and increased pulmonary vascular resistance. Furthermore, the JAK/STAT pathway has been reported to be overactivated in the pulmonary arteries of patients with PH. There are a few reviews on the JAK/STAT pathway in PH [6]. Therefore, this review aims to analyze, in depth, the expression, activation, and molecular and cellular effects of JAK/STAT activation in PH, as well as the current development of new targeted therapies for PH.

**Table 1:** Clinical classification of pulmonary hypertension (PH) [7].

**1. Pulmonary Arterial Hypertension**
1.1. Idiopathic 1.2. Heritable 1.2.1 BMPR2 mutation 1.2.2 Other mutations 1.3. Drugs and toxins induced 1.4. Associated with: 1.4.1. Connective tissue disease 1.4.2. Human immunodeficiency virus (HIV) infection 1.4.3. Portal hypertension 1.4.4. Congenital heart disease 1.4.5. Schistosomiasis 1.5 PAH long-term responders to calcium channel blockers 1.6 PAH with overt features of venous/capillaries (PVOD/PCH) involvement 1.7 Persistent PH of the newborn syndrome
**2. PH due to Left Heart Disease**
2.1. PH due to heart failure with preserved LVEF 2.2. PH due to heart failure with reduced LVEF 2.3. Valvular heart disease 2.4. Congenital/acquired cardiovascular conditions leading to post-capillary PH
**3. PH due to Lung Disease and/or Hypoxia**
3.1. Obstructive lung disease 3.2. Restrictive lung disease 3.3. Other lung disease with mixed restrictive/obstructive pattern 3.4. Hypoxia without lung disease 3.5 Developmental lung disorders
**4. PH due to Pulmonary Artery Obstructions**
4.1. Chronic thromboembolic PH 4.2. Other pulmonary arteries OBSTRUCTIONS 4.2.1. Sarcoma or angiosarcoma 4.2.2. Other malignant tumors Renal carcinoma Uterine carcinoma Germ cell tumors of the testis 4.2.3 Non-malignant tumors Uterine leiomyoma 4.2.3. Arteritis without connective tissue disease 4.2.4. Congenital pulmonary arteries stenosis 4.2.5. Parasites Hydatidosis
**5. PH with Unclear and/or Multifactorial Mechanisms**
5.1. Hematological disorders: chronic hemolytic anemia, myeloproliferative disorders 5.2. Systemic and metabolic disorders: sarcoidosis, pulmonary Langerhans cell histiocutosis, Gaucher disease, neurofibromatosis. 5.3. Others: chronic renal failure with or without hemodialysis, fibrosing mediastinitis. 5.4. Complex congenital heart disease

## 2. Expression and Distribution of the JAK/STAT Isoforms

The JAK/STAT signaling pathway regulates cell development, differentiation, proliferation, apoptosis, etc. It is involved in orchestrating the immune system to play a coordinated symphony. There are four proteins that belong to the JAK family: JAK1, JAK2, JAK3, and Tyk2. JAK1–2 and TYK2 are ubiquitous, whereas JAK3 seems to act in a hematopoietic lineage and plays critical roles in lymphocyte function. The human STAT family comprises seven isoforms (STAT1–4, 5A, 5B, and 6) [8]. The expression of some isoforms of JAK and STAT is increased in the pulmonary arteries of patients with PH (Table 2) or the pulmonary arteries of preclinical PH models (Table 3).

**Figure 1:**
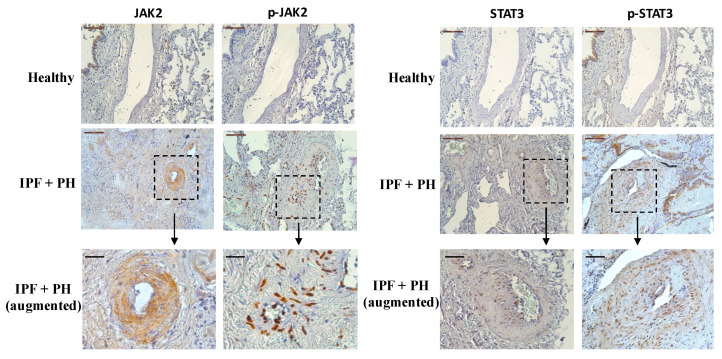
Expression and localization of JAK2/p-JAK2 and STAT3/p-STAT3 in pulmonary arteries from patients with pulmonary hypertension (PH), associated with idiopathic pulmonary fibrosis (IPF), according to immunohistochemistry. Pulmonary arteries were isolated from control subjects (*n* = 10), and IPF + PH (*n* = 9) was obtained. Scale bar: 100 μm. Scale bar (augmented): 25 μm.

The JAK2 and STAT3 mRNA transcript levels and protein expression were increased in the isolated pulmonary arteries of idiopathic fibrosis pulmonary (IPF) patients with PH. Lung section immunohistochemistry showed weak JAK2 and STAT3 expression in healthy, nonsmoking subjects, mainly localized in alveolar macrophages but not detected in pulmonary arteries. By contrast, lung sections from IPF+PH patients showed elevated expression of JAK2 and STAT3, localized in the intima and media but not in the adventitia of small pulmonary arteries (Figure 1) [9]. 

In an animal model of bleomycin-induced lung fibrosis and PH, p-JAK2/p-STAT3 were overexpressed and localized in pulmonary arteries [9].

Elevated expression of p-STAT3 has been reported in PH patients. Tyr705 STAT3 phosphorylation has been observed in human pulmonary artery endothelial cells (HPAEC) and human pulmonary artery smooth muscle cells (HPASMCs) from iPAH [10,11,12]. In addition, lung tissue sections from iPAH patients showed high expression of p-STAT3 in the endothelium, in plexiform lesions, in concentric intimal lesions, and also in the endothelium of small arteries [10]. P-STAT3 is localized in the cell nucleus of PAH-HPASMCs [13], suggesting a transcription factor role for STAT3; similar results have been found for p-JAK2, although the role of p-JAK2 as a transcription factor is lesser known [9,14,15]. P-STAT3 and p-JAK activate a broad range of transcription factors and proteins, all implicated in the regulation of proliferation and resistance to apoptosis, which leads to the development of PAH. 

According to the literature, the participation of STAT1 in PH is limited. Only one study has detailed the overexpression of STAT1 in HPASMCs from iPAH [12], but there is no evidence of a role for STAT1 in cellular function or mechanisms that induce PH. Data have shown that p-STAT3 and, to a lesser extent, STAT1 are involved in the pathophysiology of iPAH, given that STAT3 has multiple downstream targets, which increase cell survival and proliferation and inhibit apoptosis.

JAK1 is differentially ubiquitinated in hypoxic mice. The dysregulation of ubiquitination may play a role in PH, but this is not currently established [16]. Moreover, PH, induced by hypoxia, increases JAK1 mRNA expression in rats. In this model, the histochemical staining of JAK1 was observed in alveolar and bronchial epithelial cells, and in inflammatory cells from hypoxic rats [17].

The overexpression of JAK1, JAK2, JAK3, p-STAT1, and p-STAT3 at the mRNA and protein levels has also been shown in HPASMCs under hypoxic conditions [18]. The association of JAK/STAT pathway activation with other forms of PH is currently unknown; future research will be needed to clarify the role of the JAK/STAT pathway in other PH clinical entities.

The WHO classification of PH includes myeloproliferative disorders as diseases associated with PAH development [19], and, according to the literature, PH affects around 30% of patients with myelofibrosis (MF) [20,21]. In myelofibrosis (MF), an upregulation of JAK/STAT signaling has been observed. This was discovered by identifying the somatically acquired JAK2^V617F^ mutation. JAK2^V617F^ disrupts the autoinhibitory JH2 pseudokinase domain, leading to the constitutive activation of JAK2 kinase activity and STAT-mediated activation of transcription [22]. Although JAK2 is activated in MF, it is not known if this activation is the cause of the development of PH.

**Table 3:** Expression and distribution of the JAK/STAT isoforms in animal models and in vitro.

JAK/STAT Isoform	Expression and Localization in Animal Models and In Vitro
JAK1	Overexpression of JAK1 mRNA in pulmonary tissues in PH rats induced by hypoxia [17] and in hypoxic HPASMCs [18]. Histochemical staining of JAK1 in alveolar and bronchial epithelial cells, and inflammatory cells [17].
JAK2	Transcript levels and protein expression are increased in rats with PH induced by bleomycin. Localized in endothelial layer of bleomycin-treated rats [9].
STAT1	Overexpressed in iPAH-HPASMCs [12].
STAT3	Transcript levels and protein expression were increased in rats with bleomycin-induced PH [9].
	Overexpression of p-STAT3 in iPAH HPAECs and iPAH-HPASMCs [10,11,12,13].

Abbreviations: HPAEC: human pulmonary artery endothelial cells; iPAH: idiopathic pulmonary arterial hypertension; JAK: Janus kinase; HPASMCs: human pulmonary artery smooth muscle cells; STAT: signal transducer and activator of transcription.

## 3. JAK/STAT Activators

**Figure 2:**
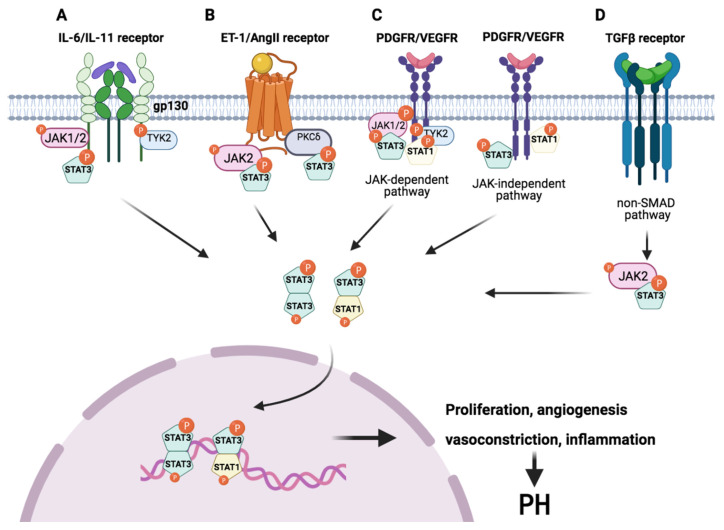
The different activators of JAK/STAT that play an important role in the pathophysiology of PH. (**A**) The binding of the cytokine (IL-6 or IL-11) to its unique receptor (IL-6R or IL-11R) triggers the homodimerization of GP130. This results in the phosphorylation of JAK1, JAK2, and Tyk2, which phosphorylate intracellular tyrosine residues that serve as docking sites for STAT3 in PH. (**B**) ET1 and Ang II exert their action through the activation of receptors that belong to a large family of transmembrane guanine nucleotide-binding protein-coupled receptors (GPCRs). JAK and STAT are constitutively expressed and directly coupled to this receptor. The binding of ETI/Ang II induces the phosphorylation of tyrosine in the JAK2 kinases, which in turn activates STAT1 and STAT3. In addition, ET-1 can stimulate the activity of PKC δ, which phosphorylates STAT3. (**C**) VEGFR and PDGFR are tyrosine kinase receptors. Ligand binding induces receptor dimerization (homo- or heterodimers) and the activation of the kinase domain. The activation of STAT1 and STAT3 can be mediated by JAK or TYK2 (the JAK-dependent pathway) or through the intrinsic tyrosine kinase activity of growth factor receptors (the JAK-independent pathway). (**D**) Stimulation with TGFβ induces the phosphorylation of JAK2 and thereby activates STAT3, but the exact mechanism is still unknown. Activated STAT1 and STAT3 translocate to the nucleus, where they bind to cis-inducing elements and promote the transcription of early growth response genes, which leads to proliferation, angiogenesis, vasoconstriction, inflammation, and resistance to apoptosis, triggering PH. Abbreviations: Ang II: angiotensin II; ET1: endothelin 1; IL-6: interleukin 6; IL-11: interleukin 11; JAK: Janus kinase; PDGF: platelet-derived growth factor; PKC δ: protein kinase Cδ; STAT3: signal transducer and activator of transcription 3; TGFβ: transforming growth factor-beta; VEGF: vascular endothelial growth factor. Created with BioRender.

The JAK/STAT pathway is crucial in transmitting signals from many cytokines and growth factors into the nucleus, regulating gene expression, and cellular functions. Cytokines of the IL-6 family, growth factors, and vascular tone mediators activate the JAK/STAT signaling pathway in a variety of cell types [23] (Table 4). JAK activation in response to a ligand involves auto- or cross-phosphorylation and leads to the phosphorylation of the receptors and to the recruitment/activation of subsets of STATs, presumably through their SH2 domains [24] (Figure 2).

### 3.1. Cytokines as Activators of JAK/STAT Pathway

Many previous studies have described the important role that interleukins of the IL-6 family play in the pathogenesis of PH, among which IL-6 and IL-11 are the most relevant. The first demonstration of increased IL-6 dates to 1996 [25]; however, the involvement of IL-11 in PH was recently demonstrated by our group [26]. The formation of a hexameric complex of IL-6/IL-6Rα/GP130 or IL-11/IL-11Rα/GP130 is required for IL-6 or IL-11 signaling, respectively. The binding of the cytokine (IL-11 or IL-6) to its unique α-receptor (IL-11R or IL-6R) triggers the homodimerization of GP130. This results in the phosphorylation of Janus kinases, which phosphorylate intracellular tyrosine residues that serve as docking sites for transcription factors such as STAT3 or the phosphatase SHP2. JAK1, JAK2, and Tyk2 have been shown to be associated with GP130, but the participation of JAK3 has not been described. Only JAK1 is essential for IL-6 and IL-11 signaling, while JAK2 and Tyk2 modulate the signals [24,27,28] (Figure 2A).

Distal pulmonary arterioles isolated from the lung tissue of patients with iPAH displayed increased levels of membrane-bound IL-6R in the smooth muscle layer compared with control arteries [29]. Patients with iPAH exhibit increased IL-6 serum levels, which correlate with their prognoses [25,30,31]. Moreover, it has been shown that the IL-6 concentration increased in HPASMCs but not endothelial cells, confirming previous work suggesting that HPASMCs may be a source of the increased IL-6 [32].

In the lungs, IL-11Rα is highly expressed in cells such as fibroblasts, vascular smooth muscle cells, and epithelial cells [33,34,35]. We have recently described that IPF patients with PH have a greater elevation of IL-11 and IL11-Rα expression in pulmonary arteries, which is associated with pulmonary artery remodeling [26]. 

IL-15 also participates in different process of PH and promotes cell survival. This cytokine was higher in the supernatant of iPAH cells than in that of control cells. IL-15 also induces the tyrosine phosphorylation of STAT3 and activates target genes involved in endothelial cell survival and proliferation [36,37]. However, there is no direct relationship of the IL-15/STAT signaling pathway with PH.

**Table 4:** Activators of JAK/STAT in PH.

Activators of JAK/STAT in PH	JAK/STAT Isoforms	Expression of JAK/STAT in Different Groups of PH
IL-11, IL-6 [24], PDGF [38], ET-1 [39]	JAK1	PAH, PH-CLD, PAH-CHD
IL-11, IL-6 [24], TGF β1 [9], PDGF [38], ET-1, Ang II [40]	JAK2	PAH, PH-CLD, PAH-CHD
IL-11 IL-6 [24], PDGF [38], Ang II [41]	Tyk2	PAH
PDGF [38], VEGF [42], Ang II [41]	STAT1	PAH
IL-11, IL-6 [24], TGF β1 [9], PDGF, VEGF [42], ET-1 [39], Ang II [43], IL-15 [37]	STAT3	PAH, PAH-CHD, PH-CLD.

Abbreviations: Ang II: angiotensin II; ET1: endothelin 1; IL: interleukin; JAK: Janus kinase; PDGF: platelet-derived growth factor; PAH: pulmonary arterial hypertension; PAH-CHD: PAH associated with congenital heart disease; PH-CLD: pulmonary hypertension associated with chronic lung disease; STAT3: signal transducer and activator of transcription 3; TGFβ: transforming growth factor-beta; VEGF: vascular endothelial growth factor.

### 3.2. Imbalance in Mediators of Vascular Tone

Hypoxic vasoconstriction is one of the characteristics of PH and is due, among other factors, to endothelial dysfunction. Endothelial dysfunction leads to a chronically impaired production of vasodilators such as NO and overexpression of vasoconstrictors, such as endothelin (ET-1) and angiotensin II (Ang II) [44].

A key clinical finding in PAH research was the reduced expression of endothelial nitric oxide synthase (eNOS) and a deficiency of NO in the lungs of patients with pulmonary hypertension [45,46,47,48]. On the other hand, elevated plasma ET-1 levels have been detected in diverse forms of PH such as PH associated with chronic lung disease or PAH-CHD [49], and correlated with disease severity [50].

Previous studies suggest that JAK1/STAT3 and JAK2/STAT3 are activated by ET-1. ET-1 increases the airway smooth muscle mass found in PH by inducing the hypertrophy and inhibiting the apoptosis of smooth muscle cells [39,40]. Another study suggests that ET-1 stimulates the activity of protein kinase Cδ (PKC δ), which phosphorylates STAT3 (Figure 2B), increasing its binding to the eNOS gene promoter and attenuating eNOS transcription and NOS signaling [51]. Hence, JAK1, JAK2, and STAT3 play a key role in the regulation of eNOS expression, which plays an important role in vascular hemostasis.

The Ang II peptide is the effector molecule of the renin–angiotensin–aldosterone system (RAAS). Ang II has been implicated in the maladaptive right ventricular hypertrophy and fibrosis associated with PH. All the hemodynamic effects, including vasoconstriction, are mediated through a single class of cell-surface receptors known as Ang II type 1 receptors (AT1Rs) [41]. In previous studies, it has been shown that the expression of Ang II and AT1R is significantly increased in patients with PAH [52,53]. ET1 and Ang II exert their actions through transmembrane guanine nucleotide-binding protein-coupled receptors (GPCR), seven transmembrane domain receptors that interact with a heterodimeric G-protein after stimulation by ligands. G-proteins consist of a GDP/GTP-binding α-subunit and a βγ-subunit complex [6]. In addition, JAK and STAT are constitutively expressed and can directly couple to AT1R [41,43]. Therefore, Ang II induces the phosphorylation of tyrosine in the kinases JAK2 and Tyk2, which in turn activates STAT1 and STAT3. Activated STAT1 and STAT3 translocate to the nucleus, where they bind to cis-inducing elements and promote the transcription of early growth response genes (Figure 2B) [23]. The blockade of JAK2 by AG-490 attenuates Ang II-induced hypertension in mice and decreases the vascular dysfunction and pulmonary arterial remodeling associated with PH [41,43,54].

### 3.3. Growth Factors as Activators of the JAK/STAT Pathway

Many growth factors have been identified to play an important role in PH. Their binding to their cell receptors promotes the activation of transmembrane cell surface tyrosine kinases (RTKs), which contribute to the proliferative, transforming, and morphogenic processes involved in the pathogenesis of PAH.

Platelet-derived growth factor (PDGF) contributes to intimal and medial vascular remodeling [55,56]. PDGFA, PDGFB, PDGFRα, and PDGFβ are overexpressed in the pulmonary arteries of PAH patients compared with healthy patients [57]. PDGF signaling is an important pathway in the abnormal pathophysiology of HPASMCs and promotes the migration of these cells from the media to intima [58]. Thus, PDGF suppression is considered a focal point in the treatment of vascular proliferative disorders [59]. PDGF stimulates the phosphorylation of the three ubiquitously expressed JAKs: JAK1, JAK2, and Tyk2. However, none of these are required for the activation of STAT1 and STAT3 by PDGF. The PDGF-induced phosphorylation of both JAK and STAT requires the intrinsic kinase activity of PDGFR, suggesting that both the JAK activation mechanism and its function, if any, differ for cytokine receptors (Figure 2C) [12,38,60].

The signaling of vascular endothelial growth factor (VEGF) is also mediated by the STAT pathway. VEGF has an anti-apoptotic and angioproliferative role in HPAECs [61]. Its biological effect is regulated by two tyrosine kinase receptors (VEGFR1 and VEGFR2). High levels of VEGF and VEGFR2 have been observed in PAH and PAH-CHD patients’ lung samples [62,63,64,65]. STAT1 and STAT3 are phosphorylated and activated after the addition of VEGF. The activation of STAT1 and STAT3 can be mediated by JAK or through the intrinsic tyrosine kinase activity of growth factor receptors. However, the phosphorylation of JAK1, JAK2, and Tyk2 has not been detected after the addition of VEGF, suggesting that the STAT activation is induced by the intrinsic tyrosine kinase activity of VEGFR [42,66] (Figure 2C).

Transforming growth factor-beta (TGFβ) regulates cellular growth, proliferation, and differentiation and is involved in angiogenesis and inflammatory processes [67]. TGFβ has also been implicated in vascular remodeling in PH, as highlighted by numerous articles [68,69,70,71]. Its expression is correlated with physiologic alterations of the pulmonary vasculature and right ventricle pressure [72]. The serum and lung tissue expression of TGFB1 is elevated in patients with iPAH and PH-CLD [73]. JAK2 and its downstream mediator, STAT3, have been identified as intracellular mediators of the profibrotic effects and pulmonary artery remodeling in fibroblasts and endothelial and smooth muscle cells. Stimulation with TGFβ induces the phosphorylation of JAK2 and thereby activates STAT3 [9,74] (Figure 2D). Moreover, it was shown that JAK2 mediates the TGFβ-induced pulmonary artery endothelial-to-mesenchymal and smooth-muscle-cell-to-myofibroblast transitions, which participate in arterial remodeling [9].

## 4. JAK/STAT Pathway and Cellular and Molecular Dysregulation in Pulmonary Hypertension

Complex vascular remodeling processes are the substrate and hallmark of pulmonary hypertension. As described above, JAK/STAT are activated in response to cytokines, growth factors, or vascular contractile agonists such as ET-1 and Ang II. The secretion of these factors is altered in PH. In the early stages of the disease, HPAECs are injured, which alters their function as a barrier. On the other hand, PASMCs are in direct contact with these factors, thus enhancing pathways of growth, resistance to apoptosis, and migration.

### 4.1. JAK/STAT Pathway and Vascular Remodeling

**Figure 3:**
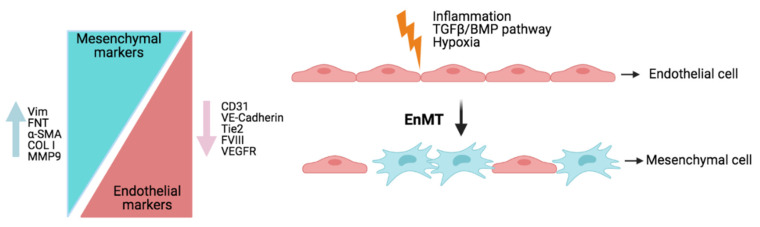
Endothelial-to-mesenchymal transition (EnMT). EnMT in PH is thought to be an important process contributing to vascular remodeling. Activated by hypoxia, inflammation, and TGF-β/BMP pathway signaling, pulmonary endothelial cells (HPAECs) undergo a cellular transition to a mesenchymal phenotype, in which they lose endothelial markers and gain mesenchymal markers. Abbreviations: COL I: collagen type 1; EnMT: transition from endothelial phenotype to a mesenchymal/myofibroblast phenotype; FNT: fibronectin; MMP9: matrix metallopeptidase 9; Vim: vimentin; α-SMA: alpha smooth muscle actin. Created with BioRender.

All forms of pulmonary hypertension are characterized by cellular and structural changes in the walls of pulmonary arteries. Our group explored the effects of JAK2 on pulmonary artery remodeling and studied the mesenchymal transition of HPAECs and HPASMCs. Incubating the HPAECs with TGFβ changed their endothelial phenotype to a mesenchymal/myofibroblast phenotype (EnMT), characterized by a loss of the endothelial markers VE-cadherin, VEGFR1, FVIII, and eNOS and an increase in the mesenchymal markers collagen type I and vimentin [9] (Figure 3). TGFβ1 also increased the expression of collagen type I and vimentin in HPASMCs and HPASMC proliferation. These effects were inhibited by siRNA-JAK2. Furthermore, the pulmonary arteries of IPF+PH showed coimmunostaining with α-SMA and JAK2/p-STAT3 in endothelial cells, suggesting that the endothelial cells had transformed into myofibroblasts [9]. Other studies proved that endothelial cells of intimal and plexiform lesions from PAH patients are transformed into mesenchymal cells/myofibroblasts, thus contributing to PH [75]. Recently, our group found that fibroblasts isolated from IL-11-treated mice had an endothelial origin [76]. Accumulating evidence suggests that EnMT plays a pivotal role in the initiation and progression of this disease [75,77,78] and in pulmonary artery remodeling, which contributes to the progression of occlusive neointimal lesions in pulmonary arteries [75,79].

### 4.2. JAK/STAT Pathway, Proliferation, and Resistance to Apoptosis

PAH is a vascular disease characterized by pulmonary artery smooth muscle cell proliferation and pulmonary artery hypertrophy [13]. STAT3 inhibition prevents neointimal formation by inhibiting the proliferation and promoting the apoptosis of neointimal smooth muscle cells [80]. In cancer, STAT3 promotes the expression of the provirus integration site for Moloney murine leukemia virus’ Pim1, a proto-oncogene serine/threonine-protein kinase. The development and progression of certain cancers due to increased cell proliferation and resistance to apoptosis have been related to the overexpression of Pim1 [81,82,83]. Paulin et al. showed that the treatment of healthy PASMCs with ET1, Ang II, and PDFG caused an increase in the PY705-STAT3/STAT3 ratio. Once activated, STAT3 increases Pim1 and nuclear factor of activated T cell (NFAT2) expression. They provide in vitro and in vivo evidence of the mechanisms by which Pim1 inhibition reverses PAH, which involves the inhibition of PASMC proliferation and decreasing Bcl-2, increasing apoptosis. They focused on PASMCs and not HPAECs because the latter are downregulated in established PAH, but Pim1 may also be implicated in endothelium-related vascular lesions, such as plexiform lesions [13]. The proliferation of HPAECs from iPAH was evaluated in the presence of VEGF and IL-15, and the authors demonstrated that there was greater proliferation than in control cells, especially in cells stimulated with VEGF. Moreover, cell proliferation was blocked by AG-490, a pharmacological inhibitor of the JAK/STAT3 signaling pathway. Finally, they observed that the increase in cell viability occurs in association with increased expression of the prosurvival factors Mcl-1, IL-15, and BcL-2 and persistent activation of the critical prosurvival STAT3 signal transduction pathway [10].

On the other hand, it has been suggested that STAT3 regulates the expression of miR-204 [84]. miR-204 expression in PASMCs is downregulated in both human and rodent PAH. STAT3 activation suppresses miR-204 expression, which activates the Src kinase and NFAT. STAT3 also directly induces the expression of NFATc2. NFAT and SHP2 were required to maintain PAH-PASMC proliferation and resistance to apoptosis [11]. Caspase-1 also induced the proliferation of PASMCs through the caspase-1/IL-8/IL-6/STAT3 signaling pathway, causing PH in mice exposed to hypoxia [85].

### 4.3. JAK/STAT Pathway, Migration, and Angiogenesis

Migration and angiogenesis in cells implicated in PH have not been extensively studied. Studies have identified STAT3 as the central prosurvival molecular signaling pathway and the primary regulator of angiogenesis [10]. Migration was greater in iPAH cells than in the control in response to VEGF or FGF. Other studies have indicated that dominant-negative STAT3 abolishes VEGF-induced endothelial cell migration and suppresses VEGF-induced tube formation on collagen gels [66].

It is postulated that, in lungs with PH, HPAECs in plexiform lesions express proteins involved in angiogenesis, especially VEGF. VEGF stimulates the migration and proliferation of HPAECs [63]. However, concentric proliferative lesions, which occur proximally to the plexiform lesion and then evolve distally in a thin-walled dilated blood vessel, showed reduced expression of VEGF and VEGFR2 [86].

### 4.4. Imbalance in Vasoactive Mediators: Vasoconstriction

In patients with PH, the generation of vasodilatory mediators is reduced, and the generation of vasoconstrictor mediators is increased, which in turn increases the generation of reactive oxygen species and reactive nitrogen species, which play an important role in the development and/or progression of PH [87,88,89,90]. ROS modulate the effects and/or release of several vasoactive factors, such as ET-1 and prostacyclin, which can acutely influence vessel tone [91,92]. Furthermore, the inhibition of ROS production has been shown to attenuate hypoxic PH (HPH) in both rat and mouse models of chronic hypoxia [93].

Although several enzymes produce ROS, the most important is arguably NADPH oxidase, which plays a key role in the remodeling and vasoconstrictive aspects of PH [92]. Seven enzyme subtypes of Nox have been identified in a wide range of cell types, but only Nox1, Nox2, Nox4, and Nox5 are found in the pulmonary vasculature [91,94]. Nox4 is overexpressed in PASMCs, and its siRNA-mediated silencing reduces ROS levels and cell proliferation, suggesting that Nox4 mediates pulmonary vascular remodeling [87,89,95]. Ang II plays an important role in the development of hypertension and is one of the most important inducers of NADPH oxidase-dependent superoxide production in PASMCs [96] and in the entire vascular wall [97]. The effects of Ang II on NADPH oxidases are mediated mainly through the AT1R receptor [98]. Ang II also stimulates NADPH oxidases, which are involved in intracellular H_2_O_2_ production and mediate vascular hypertrophy [95,99]. Although ATR1 activation induces the JAK2/STAT3 pathway, there is no direct evidence of an interaction between JAK/STAT and the NADPH oxidase system.

The relationship between reactive nitrogen species and JAK/STAT is more direct. As we have mentioned previously, ET-1 overexpression promotes STAT3 phosphorylation, which leads to a decrease in NO and eNOS levels. To verify the involvement of STAT3 in this process, studies were carried out with dominant negative mutants of PKCδ/STAT3, where it was observed that an increase in STAT3 activity and decrease in eNOS promoter activity were inhibited [51]. This suggests that STAT3 antagonism could provide a novel therapeutic strategy for improving vascular homeostasis.

Serotonin (5-HT), Ang II, and ET-1 promote the vasoconstriction of the pulmonary artery. Previous reports have shown that the inhibition of JAK2 can reduce the contraction of the rat aortic ring induced by intracellular Ca^2+^ and 5-HT, Ang II, and ET-1 [100,101]. In addition, there is evidence of the role of JAK2 in the pulmonary vasoconstriction of small pulmonary arteries in control subjects and patients with PH-IPF. It is known that the vascular remodeling of the human pulmonary artery occurs in small resistant-type intrapulmonary vessels that are part of the pulmonary vascular bed, which are responsible for the pressure elevation observed in PH [102]. JAK2 inhibition with JSI-124 relaxed small pulmonary arteries precontracted using 5-HT in patients with PH-IPF. Moreover, JAK2 has been suggested to play a role in the maintenance of the basal tone of the pulmonary arteries because JSI-124 had direct relaxing effects on untreated basal pulmonary arteries. Electrophysiological experiments using the patch clamp technique demonstrated that JAK2 inhibits BK_Ca_ potassium currents and increases intracellular Ca^2+^ in PASMCs, thus contributing to pulmonary arteries’ constriction [9].

### 4.5. JAK/STAT Pathway and Inflammation Associated with Pulmonary Hypertension

It is now recognized that perivascular inflammation is a common contributing factor in almost all forms of PH [103,104,105,106] because inflammatory cell infiltrates comprising T- and B-lymphocytes and macrophages have been identified. Upon endothelial cell injury, HPAECs become dysfunctional and alter their secretion of cytokines and other factors that regulate coagulation, thrombosis, and vascular tone [107]. However, the exact mechanisms by which inflammation might facilitate the progression of PH are still under investigation. Recent evidence has pointed to the role played by exogenous signaling molecules produced by inflammatory cells. The metabolite of the 15-lipoxygenase pathway, 15-HETE, upregulates several cytokines such as IL-6 and TNF-α, both of which have been implicated in PH [108,109]. IL-6 and IL-8 may be an important part of the initial response to injury, contributing to the process of vascular remodeling in PAH, and have been demonstrated to modulate HPAECs’ and HPASMCs’ function [30]. Graham et al. studied the role of the IL-6–STAT3–NFATc2 pathway and demonstrated that medial remodeling was decreased in IL-6^−/−^ mice treated with hypoxia or *Schistosoma* [110].

**Figure 4:**
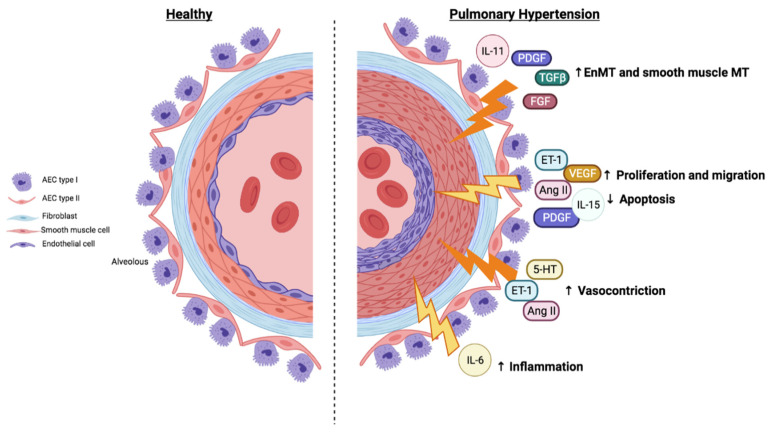
Cellular and molecular processes in pulmonary hypertension. PH is characterized by abnormal pulmonary artery remodeling, excessive pulmonary vasoconstriction, and processes that usually affect all vessel layers (intima, media, and adventitia), resulting in the loss of vascular cross-sectional area and, therefore, elevated pulmonary vascular resistance. The intimal changes include endothelial injury, endothelial and muscular cell proliferation, and the invasion of the intima by myofibroblasts, with enhanced matrix deposition and intimal fibrosis. These structural changes suggest a switch from a quiescent state to a proliferative, apoptosis-resistant cellular phenotype. These structural changes are triggered by the dysregulation of the expression and release of cytokines, growth factors, and vascular contractile agonists such as ET1 or Ang II. Abbreviations: AEC: alveolar epithelial cells; Ang II: angiotensin II; ET-1: endothelin 1; FGF: fibroblast growth factor; IL: interleukin; PDGF: platelet-derived growth factor; TGFβ: transforming growth factor-β; VEGF: vascular endothelial growth factor; 5-HT: serotonin. Created with BioRender.

We can conclude that the JAK/STAT pathway contributes to cell proliferation, differentiation, growth, and resistance to apoptosis. However, the participation of the JAK/STAT pathway in inflammation, the production of ROS, and angiogenesis has not been widely studied (Figure 4).

## 5. Therapeutic Management of JAK/STAT in PH

Alterations in kinase activity result in the dysregulation of cytokine production, proliferation, cell survival, and transcription of genes that contribute to PH [111]. As we have described, growth factors and interleukins send signals through their respective receptors and activate the JAK/STAT signaling pathway, which ultimately leads to increased proliferation, resistance to apoptosis, angiogenesis, and vasoconstriction. A range of JAK/STAT inhibitors are currently being tested in clinical trials or in preclinical models of PH (Table 5).

It is known that about 30% of patients with myelofibrosis [20,112] develop PH [113]. Ruxolitinib is a treatment approved by the FDA for treating myelofibrosis [114,115] in patients with a positive JAK2^V617F^ mutation and has been reported to improve PH [21,112]. The effects of ruxolitinib in 15 patients with PH associated with MF were evaluated, and it was concluded that the treatment improved echocardiographic findings, resulting in significant reductions in right ventricular systolic blood pressure, in 66% of patients. In addition, ruxolitinib led to an increase in the plasma levels of nitric oxide in 46% of the patients, and most of the patients had reductions in the levels of serum biomarkers of pulmonary hypertension, including the N-terminal prohormone of brain natriuretic peptide (73%), von Willebrand antigen (86%), ristocetin cofactor activity (73%), and uric acid (60%) [21,112].

Moreover, a therapeutic benefit from JAK2 inhibition by ruxolitinib has been demonstrated in preclinical models of PH. In peptide-based kinase activity screening, JAK2 was identified as highly active in PASMCs from PAH patients compared to healthy cells. The inhibition of JAK2 by ruxolitinib led to an improvement in cardiopulmonary function via decreases in right ventricular systolic pressure (RVSP) and the pulmonary vascular resistance index (PVRI), and restored the cardiac index (CI) in animal models [116]. Furthermore, ruxolitinib inhibits the development of PH and partially reduced right ventricular hypertrophy in two independent animal models of PAH (a monocrotaline rat model and chronic hypoxia mouse model) through the blockade of the JAK2–STAT3 signaling pathway [116]. However, the clinical use of ruxolitinib in other PH forms has not been tested, probably due to the high rate of adverse side effects [117].

XL019, CEP701 (lestaurtinib), and TG101348 (fedratinib) are other pan-JAK inhibitors in an early clinical phase of development for the treatment of MF [117]. To our knowledge, there are no preclinical or clinical trials of treatment with XL019 and CEP-701 in patients or models of PH. Nevertheless, the blockade of JAK2 by fedratinib in hypoxic HPASMCs has been studied, and we can conclude that this drug suppressed hypoxia-induced HPASMC proliferation and attenuated pulmonary vascular remodeling in vitro [118].

On the other hand, regarding STAT3 inhibitors, there is evidence that dehydroepiandrosterone (DHEA) is an efficient STAT3 inhibitor. The exact molecular mechanism by which DHEA decreases STAT3 activation remains to be established. Two clinical trials are being conducted with DHEA for chronic obstructive pulmonary disease and pulmonary hypertension (PH–COPD), and there are two other clinical trials for PAH. According to the study registered as NCT00581087, treatment with DHEA significantly improves the 6-MWT distance, pulmonary hemodynamics, and DLCO in patients with PH associated with COPD, without worsening gas exchange (as occurs in other pharmacological treatments of HP) [119].

To our knowledge, these are the only JAK/STAT inhibitors in clinical development. However, other inhibitors of the JAK/STAT pathway are being tested in preclinical trials in vitro and in vivo, as described below.

Our group showed that Jak2 inhibition by JSI-124 ameliorates pulmonary artery remodeling, increases pulmonary artery relaxation, and improves the overall symptoms of the disease in this experimental model of bleomycin-induced IPF with PH [9].

Plumbagin (PLB) is a natural organic compound known to block STAT. In vitro findings indicate that the inhibition of the STAT3/NFAT axis by PLB decreases proliferation and increases apoptosis in PASMCs from PAH. In vivo, the oral administration of PLB decreases distal pulmonary artery remodeling, mean pulmonary artery pressure, and right ventricular hypertrophy without affecting the systemic circulation in rats with monocrotaline- or chronic-hypoxia-induced PAH [120]. Nonetheless, little is known about the human tolerance of the molecule and its side effects, making its translation to clinical use unlikely.

Several JAK/STAT inhibitors are in early-phase clinical trials for the treatment of diverse diseases [6]. This might facilitate their utilization in PAH treatment, and data on their tolerability and efficiency will be available soon.

**Table 5:** Therapeutic management of JAK/STAT in PH.

Therapy	Target (IC50)	Groups of PH	Status
Ruxolitinib	JAK1 (2,7nM), JAK2 (4,5nM), JAK3 (332nM) TYK2 (19nM)	PH-MF	Approved [114,115]
		Pre-clinical PH models	Pre-clinical models of PH [116]
XL019,	JAK1 (130nM), JAK2 (2nM), JAK3 (250nM), TYK2 (340nM)	PH-MF	Discontinued [117]
CEP701 (lestaurtinib)	JAK2 (0,9nM), FLT3 (3nM), TrkA (4nM)	PH-MF	Phase I/II [117]
TG101348	JAK1 (105nM), JAK2 (3nM), JAK3 (1000nM) TYK2 (405nM)	PH-MF	Phase II [117]
		PAH	In vitro [118]
JSI-124	JAK2, STAT3 (500nM)	Pre-clinical PH models	Bleomycin model [9]
DHEA	STAT3	PH-COPD	Phase III [121]
Plumbagin	STAT3	Pre-clinical PH models	Monocrotaline and chronic hypoxia model [120]

Abbreviations: PAH: pulmonary arterial hypertension; JAK: Janus kinase; PH–COPD: pulmonary hypertension associated with chronic obstructive pulmonary diseases; PH–IPF: pulmonary hypertension associated with idiopathic pulmonary fibrosis; PH–MF: pulmonary hypertension associated with myelofibrosis; STAT: signal transducer and activator of transcription. IC_50_ values were extracted from [122,123]. IC_50_ data for DHEA and plumbagin are not available.

## 6. Conclusions

PH is a disease that affects the pulmonary vasculature, increasing pulmonary vascular resistance and pulmonary pressure, which leads to compensatory right ventricular hypertrophy, which can turn into right ventricular failure. The current approved therapies are limited, despite the improvement of quality of life that could be realized. They remain insufficient for reversing PH and improving survival. Every day, there is more evidence of the participation of JAK and STAT as mediators in the pathology of PH. In addition, preclinical and clinical trials reveal that they may be promising therapeutic targets. As we described above, JAK and STAT participate in proliferation, survival, migration, inflammation, and vasoconstriction, which lead to PH pathology. Targeting the JAK/STAT pathway has the potential to decrease these hallmarks, and JAK/STAT inhibition could be promising in patients with PH.

## Figures and Tables

**Table 2:** Expression and distribution of the JAK/STAT isoforms in human tissues.

JAK/STAT Isoform	Groups of PH	Expression and Localization in Humans
JAK2	PH-CLD	Overexpression of transcript levels and protein in human isolated pulmonary arteries. Overexpressed in the intima and media of human small pulmonary arteries [9].
STAT3	PH-CLD	Elevated expression of STAT3 in the intima and media of small pulmonary arteries.Overexpression of transcript levels and protein in human isolated pulmonary arteries [9]
	iPAH	Overexpression of p-STAT3 in endothelium, plexiform lesions, and concentric intimal lesions in iPAH patients [10].

Abbreviations: iPAH: idiopathic pulmonary arterial hypertension; JAK: Janus kinase; PH-CLD: pulmonary hypertension associated with chronic lung disease; STAT: signal transducer and activator of transcription.
